# The influence of general knowledge test performance on self-ratings of and perceived relationships between intelligence, knowledge, and memory

**DOI:** 10.1038/s41598-023-42205-y

**Published:** 2023-09-21

**Authors:** Jennifer H. Coane, John Cipollini, Charlotte Beaulieu, Julianna Song, Sharda Umanath

**Affiliations:** 1 Department of Psychology, Colby College, Waterville, ME 04901 USA; 2 Claremont McKenna College, Claremont, USA

**Keywords:** Human behaviour, Psychology

## Abstract

The present study examined how taking a general knowledge (GK) test affects perceptions of one’s intelligence, memory, and knowledge and the relationship between these three constructs. Participants rated their abilities on each construct and the strength of the relationships between them before and after completing an easy or hard GK test or control task. In Experiment 1, participants were (mis)informed that GK questions were correctly answered by 50% of the population; in Experiment 2, no such information was provided. Regardless of (mis)information about others’ performance, participants in the Hard condition believed they had a worse memory, were less knowledgeable, and were less intelligent post-task. However, the strength of the perceived relationship between GK and intelligence decreased only when participants were misled. Judgments of one’s intelligence, memory, and knowledge can be manipulated by taking a GK test, and individuals engage in self-protective behavior to reduce the potential threat to one’s self-concept.

## Introduction

Learners of all ages need to be able to accurately assess their abilities, strengths, and areas for further development. Such metacognition is important for learning and task performance^1^. Broadly defined, metacognition involves monitoring one’s knowledge and/or performance on a task and controlling behaviors to lead to a desired outcome. Therefore, it involves the accurate assessment of one’s abilities, knowledge, and skills. Among the abilities that are central to academic success are intelligence, level of knowledge, and the ability to retrieve information when needed (i.e., memory skills). Our interest is in understanding participants’ self-assessments of these constructs (i.e., their metacognitions surrounding them), their notions of the relationships among them, and whether these perceptions are malleable to experience. In what follows, we review prior work that shows the malleability of perceptions within constructs; the novel contribution of this work is to examine how manipulating an experience with one construct (knowledge) can impact related, yet independent, constructs (memory and intelligence). In addition, we examined how perceived performance in one domain affects the degree to which individuals consider these three core cognitive constructs to be related.

In education, errors in the evaluation of one’s abilities can have practical consequences. A student who overestimates their preparation for an assessment (*monitoring*) might stop studying or reviewing (*control*^2^) and perform worse on the assessment than if they had continued their preparation. In contrast, a student who underestimates their preparation might continue to study and perform better than expected^3^. Accurate metacognitive skills are also necessary for evaluating more stable individual traits and characteristics, such as one’s intelligence, athletic prowess, or other skills. Self-worth, or what facets of the self that one deems as essential for psychological well-being, can be contingent on one or more domains of performance^4^. In any domain, social and cognitive factors can influence the accuracy of one’s assessments. In general, people strive to maintain a positive self-concept^5,6^. When perceiving a threat to one of the domains or contingencies central to self-worth, individuals might engage in defensive or avoidant measures (e.g., distancing, blaming, or reappraisal^6^). For example, a student for whom academic performance is important might attribute a poor grade to an unfair assessment.

## Factors affecting self-assessments and their consequences

As noted, subjective evaluations of abilities are not fixed, and many factors can affect them. Expectations shape and modify objective performance across domains, from placebo effects in medical and therapeutic contexts, to stereotype threats, to Hawthorne and Pygmalion effects^7^. If threats to the self-concept or self-worth are perceived, regulatory behaviors or shifts in beliefs or attitudes might ensue. A comparative or evaluative context might trigger such measures. Most relevant to the present work, expectations of ease versus challenge in answering (actually difficult) general knowledge questions can affect participants’ reporting of retrieval failures like tip-of-the-tongue states. That is, the incidence of tip-of-the-tongue states reported increased when participants expected the questions to be easy and were presumably under pressure to perform well, despite actual performance being the same^8^. Experience can also be shaped by feedback, direct or indirect. When feedback yields a discrepancy between performance and a goal state, individuals may reallocate resources to maintain their self-concept^9^. Feedback can also affect subsequent performance, goals, and evaluations of one’s abilities^10,11^. Negative feedback might lead participants to justify their real or perceived failure, by engaging in various protective mechanisms, from self-handicapping (e.g., blaming subsequent failure as a lack of effort rather than ability) to framing a situation more positively^12^.

Of particular interest in the present work are findings related to assessments of one’s memory, knowledge, and intelligence. For example, a difficult task can cause participants to shift from over- to under-confidence^13^, and perceived difficulty or ease during learning can affect metacognitive assessments^14,15^, even when not affecting actual performance. Recent experiences affect metacognitive control: experience of success, such as what an easy task would afford, compared to experiences of failure, caused by difficult tasks, influenced what type of task participants engaged in subsequently^16^. Thus, subjective evaluations of ability and measurable performance can be influenced by empirical manipulations, expectations, biases, and recent experience.

Thus, perceptions of general cognitive abilities, such as intelligence, as well as judgments of task performance, are flexible and affected by context. Students who believe that intelligence is a fixed, heritable trait show more helpless attitudes about their academic abilities and downward academic trajectories relative to students who believe intelligence is a malleable trait^17^. People may change their beliefs about their intelligence if given a cognitive task at which they excel. Thus, beliefs about what makes someone intelligent likely depend on many factors, such as performance on other tasks^18,19^, feedback^12,20^, and education.

## The present work

Although a substantial amount of work has examined lay theories of intelligence^21,22^, and some work has explored lay theories of memory and knowledge^23–25^, there is a gap in understanding how lay participants perceive these constructs to be related to one another, both implicitly and explicitly^26^. When explicitly asked to define these constructs, lay participants indicated that knowledge and intelligence are strongly related, albeit asymmetrically: when defining what *being intelligent* meant, participants frequently referred to knowledge (i.e., crystallized intelligence^27^) but they rarely referred to intelligence when defining what *knowing* meant^26^. Memory, operationally defined by asking participants what *remembering* meant, emerged as distinct in terms of core dimensions from both *knowing* and *being intelligent.* We note that expert definitions of these constructs are varied and complex; however, it is beyond the scope of this work to directly compare expert to lay conceptions.

The questions we addressed here were: (1) How do expectations and experience affect self-assessments of memory, knowledge, and intelligence? (2) To what extent do lay participants consider memory, knowledge, and intelligence to be interrelated? And (3) To what extent are the perceived relationships fixed or malleable? Given that recent experience affects participants’ choices of tasks to engage with^16,28^ and estimates of their abilities^29^, experiencing difficulty in retrieving information might cause individuals to shift how they perceive their abilities and these constructs. Specifically, we manipulated how attempting to retrieve information from the knowledge base—and failing or succeeding—affected these measures. We selected a knowledge task for a few reasons: first, there are performance norms, allowing us to identify questions that would elicit effort and probable failure or ease and probable success; second, attempting to retrieve information from semantic memory, by engaging retrieval processes for a recognition task, might affect participants’ evaluations of their memory; and, third, knowledge is a core component of crystallized intelligence. Metacognitive accuracy tends to be higher when to-be-learned material is meaningful and related to prior knowledge^30^, suggesting knowledge and memory are involved in metacognitive judgments. Furthermore, fluid intelligence is moderately correlated with metacognitive skills^31^, and, given the strong relationship between *knowing* and *being intelligent*^26^, perceived performance on a general knowledge task was likely to impact perceived intelligence. Thus, general knowledge questions might affect perceptions of all three constructs under investigation.

People’s beliefs about the *relations* between knowledge, intelligence, and memory may also depend on the extent to which they excel in these cognitive domains^19^. For example, poor performance on a knowledge task may lead one to dismiss GK as an indicator of intelligence because this conceptualization of intelligence would challenge their positive self-concept. Conversely, if one performs well, they may increase their belief in the notion that GK is indeed a valid indicator of intelligence, as this performs a self-enhancing function. In addition, people may conceptualize memory as being synonymous with GK—being able to recall the relevant information. In sum, bottom-up experiences with task difficulty might influence the top-down information about participants’ own abilities and result in a re-evaluation^18^.

Participants completed an easy or hard GK test, and we measured their self-assessments of their knowledge, intelligence, and memory and the relationship between these three constructs before and after test completion. In Experiment 1, participants were falsely told that half of the general population could answer the questions correctly, to ensure that those in the Easy GK condition would believe they excelled at the test whereas those in the Hard GK condition would believe they performed poorly. In Experiment 2, there was no such (mis)information about population-level performance. In both experiments, a control group completed an unrelated task to assess baseline changes in ratings. Note that this is different from previous work^8^, in which participants were given the same set of questions but some were told to expect an easy set of GK questions whereas others were told to expect a difficult set. Here, the questions themselves were actually selected to be easy versus hard. Thus, in Experiment 1, we were able to examine the effect of congruence versus incongruence between the experience of taking the test and then the expectations about performance with relation to one’s own abilities on perceptions of the relationships between intelligence, memory, and knowledge. In Experiment 2, we were able to examine the direct influence of the actual experience of ease versus struggle in recognizing correct answers on perceptions of the relationships between intelligence, memory, and knowledge.

We predicted that participants who completed the hard GK test would rate themselves less favorably after the test, especially when provided (mis)information about population-level performance. This would reflect an accurate reassessment of one’s abilities. Participants performing poorly on the hard GK test might also rate their memory poorly, if the failure to recognize the correct answer was accompanied by effort. Finally, we predicted that participants who received the hard GK test would believe that knowledge and intelligence are less strongly related because of the potential threat to their self-image. In sum, we predicted that a discrepancy between a positive dimension of self-worth and actual performance would lead participants to engage in corrective or protective measures to preserve their self-concept^6, 10^. Of particular interest was the fact that the difficulty manipulation was directly relevant for only one of the three constructs under examination here (knowledge). Whether experience-based feedback in one domain would generalize to evaluations of ability in other general cognitive domains would extend previous findings showing shifts in evaluations within the targeted domain^29^.

## Experiment 1

### Method

#### Participants

Two hundred thirteen participants were recruited for an online survey using Prolific (http://www.prolific.co), an online data collection tool where participants can complete studies for monetary compensation. The target sample size was 65 participants in each between-subjects condition. We aimed to recruit a sample comparable to that employed in previous research examining lay understandings of memory constructs^25,32^. A sensitivity analysis in G*Power^33^ indicated that a sample of 200 participants with a correlation between repeated measures of 0.75 would be sufficient to detect an effect size *f* of 0.10. Therefore, the sample would suffice to detect a small effect.

Participants were at least high school graduates (average was 14.31 years of education), between 18 and 30 years old, and required to have a US IP address to ensure that participants’ conceptualizations of the constructs in question would be related to their common uses in American culture, and they would have a similar level of GK. Six participants who opened the survey revoked consent and five timed out, leaving 202 data sets (121 women; three unidentified). The average age was 22.72 (*SD* = 3.23, range 18–32). The average completion time was 7.54 min (*SD* = 5.03, range 1.40–38.13), and participants were compensated at an average rate of $11.71 USD per hour.

Both studies were approved by the Institutional Review Board at Colby College and adhered to the ethical guidelines of the American Psychological Association.

#### Materials and procedure

Participants clicked on a link to the online survey. The survey was programmed and conducted via Qualtrics (Qualtrics, Provo, UT) and participants provided informed consent at the outset. The first section included three open-ended questions where participants were instructed to type their responses: (1) “What does ‘remembering’ mean to you?”, (2) “What does ‘knowing’ mean to you?” and (3) “What does ‘intelligence’ mean to you?” The coding of these questions is not reported here.

The second section included two sets of Likert-scale questions. The first set asked, “On a scale from 1 to 7, how knowledgeable are you?” (1 = not at all and 7 = extremely),” “On a scale from 1 to 7, how intelligent are you?” (1 = not at all and 7 = extremely), and “On a scale from 1 to 7, how good is your memory?” (1 = very poor and 7 = very strong). The second set of Likert-scale items (1 = not at all and 7 = very much) asked participants, “To what extent do you believe a general knowledge test reflects one’s intelligence?” “To what extent do you believe that having a good memory makes one knowledgeable?” and “To what extent do you believe that having a good memory makes one intelligent?” The first set of questions was always presented first, and the order within each set of questions was randomized each time. Participants responded to both sets twice: once before the experimental manipulation and once after using a slider with only the values 1 and 7 marked on it. Values were recorded with one decimal point. Sliders were used to ensure that answers in the later section would not be anchored to prior responses because participants would be unable to remember the exact numerical value their answers corresponded to.

Participants were randomly assigned to either the Easy GK (*n* = 67), Hard GK (*n* = 67), or Control condition (*n* = 68). In the Easy GK and Hard GK conditions, before completing the GK test, participants read the following: “About 50% of the general population can answer the following questions correctly.” This statement was intended to make participants in the Easy GK condition feel that they excelled at the test, given that they would likely answer most, if not all, questions correctly. Thus, they would believe they performed better than most people. Conversely, we expected participants in the Hard GK condition to feel that they struggled with the test, as they would not be able to answer most, if any, of the questions correctly. Therefore, they would believe they performed poorly relative to most people^34^. Participants were asked to answer the questions without consulting external sources.

Fifteen questions for the easy GK test were chosen from published norms^35^ and were selected because they had a 0.5 probability of recall or higher (*M* = 0.67). Similarly, 15 questions were selected from the same source for the hard GK test on the basis that they had a 0.01 probability of recall or lower (*M* = 0.00). Thus, the Easy questions were much easier than the 50% (mis)information suggested, and the Hard questions were much harder. In Tauber et al., participants answered via free recall, whereas we used questions with 4-alternative multiple-choice answers. In addition to the correct answer, three foils were included^36^. Participants were provided with immediate accuracy feedback. The order of questions was randomized for each participant.

Participants in the control group completed a task in which they ranked five unrelated words in each of 8 groups in order from most pleasant to least pleasant. This task was designed to take a similar amount of time as the GK tests.

After the manipulation, all participants responded to the same Likert-scale items they answered at the start of the survey. Then, they were instructed to input any reason they may have had for changing their answers from the first to the second time they answered them. Participants in the GK test conditions were also asked if they had searched for any answers online. All participants then viewed a debriefing form and were compensated.

### Results

#### Analysis overview

We analyzed the numerical ratings of participants for their own knowledge, memory, and intelligence and the ratings regarding the strength of the relationship between these constructs (see Table 1 for descriptive statistics). Higher scores reflect higher ratings/stronger relationships. Five participants’ data in the Control condition were excluded due to not completing the task. All participants in the GK test conditions responded “no” to the question about whether they had looked up answers to any questions online. In all analyses, we report partial eta squared as a measure of effect size for ANOVAs and Cohen’s *d* for *t*-tests, and, where relevant, degrees of freedom reflect a correction for violations of assumptions (Greenhouse–Geisser for ANOVA). When multiple comparisons were required, a Bonferroni correction was applied.

**Table 1:** Descriptive statistics for all measures in study 1 and 2.

		Range	Average	Standard deviation	Skewness	Kurtosis
Study 1	Knowledge rating pre-test	1.6–7	4.60	1.08	− 0.14	− 0.19
	Intelligence rating pre-test	1.2–7	4.81	1.06	− 0.49	0.65
	Memory rating pre-test	1–7	4.29	1.35	− 0.06	− 0.52
	Knowledge rating post-test	1–7	4.41	1.34	− 0.23	− 0.43
	Intelligence rating post-test	1–7	4.71	1.26	− 0.54	0.18
	Memory rating post-test	1–7	4.02	1.55	− 0.09	− 0.97
	GK and intelligence pre-test	1–7	3.55	1.58	0.23	− 0.82
	GK and memory pre-test	1–7	4.48	1.38	− 0.37	− 0.26
	Memory and intelligence pre-test	1–7	3.74	1.50	0.11	− 0.73
	GK and intelligence post-test	1–7	3.34	1.60	0.35	− 0.86
	GK and memory post-test	1–7	4.30	1.36	− 0.32	− 0.33
	Memory and intelligence post-test	1–7	3.63	1.49	0.15	− 0.75
	GK test performance (proportion correct)	0–1	0.59	0.36	− 0.05	− 1.81
Study 2	Knowledge rating pre-test	2.2–7	4.61	0.98	− 0.21	− 0.26
	Intelligence rating pre-test	1.7–7	4.89	1.06	− 0.36	− 0.01
	Memory rating pre-test	1.3–7	4.36	1.34	− 0.06	− 0.74
	Knowledge rating post-test	1.1–7	4.41	1.21	− 0.44	0.06
	Intelligence rating post-test	1.3–7	4.76	1.26	− 0.61	− 0.01
	Memory rating post-test	1.1–7	4.23	1.46	− 0.21	− 0.79
	GK and intelligence pre-test	1–7	3.56	1.49	0.24	− 0.52
	GK and memory pre-test	1–7	4.55	1.48	− 0.36	− 0.6
	Memory and intelligence pre-test	1–7	4.04	1.52	− 0.09	− 0.84
	GK and intelligence post-test	1–6.9	3.54	1.48	0.15	− 0.84
	GK and memory post-test	1–7	4.4	1.41	− 0.21	− 0.48
	Memory and intelligence post-test	1–7	3.92	1.43	− 0.02	− 0.8
	GK test performance (proportion correct)	0–1	0.59	0.37	− 0.11	− 1.78

Performance on the GK test confirmed that the easy questions (*M* = 0.94, *SEM* = 0.009) were correctly answered more than the hard questions (*M* = 0.25, *SEM* = 0.01), *t*(120.63) = 43.98, *p* < 0.001, *d* = 7.57.

In the analyses that follow, for each measure, we report a 2 (time of rating: pre-task, post-task) × 3 (task type: Easy GK test, Hard GK test, Control) mixed ANOVA with time of rating as a within-subjects factor and task type as a between-subjects factor.

#### Self-ratings of intelligence, knowledge, and memory

##### Overall ratings

**Figure 1:**
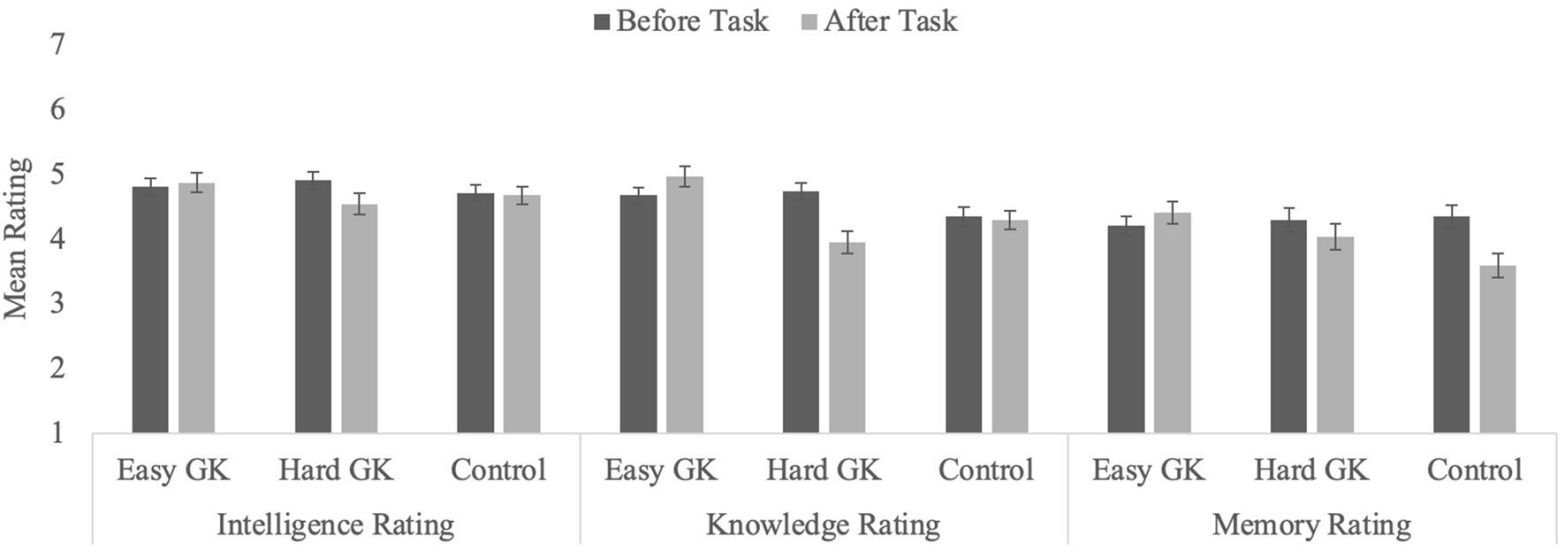
Average ratings for self-assessment of intelligence, knowledgeability, and memory in Experiment 1 as a function of condition and timing (error bars represent standard error of the mean).

Average ratings as a function of timing and task type are in Table 2 and in Fig. 1. We provide both figures and tables because we consider it to be of most help to see the numbers in the tables, note which comparisons are significant, and glean the general patterns from the figures. Pre-task, participants in all three conditions rated their knowledge and intelligence above the mid-point (4.0), all *t*s ≥ 2.58, *p*s ≤ 0.012. Self-rated memory did not differ from the mid-point (all *t*s ≤ 2.17, *p*s ≥ 0.033 [not significant following a Bonferroni correction with α = 0.017]). Across conditions, self-ratings did not differ at pre-task (all *p*s ≥ 0.094). Post-task, self-rated intelligence was still above the mid-point in all three conditions (all *t*s ≥ 3.27, *p*s ≤ 0.002), but knowledge and memory ratings were only above four in the Easy GK test condition, *t*s ≥ 2.47, *p*s ≤ 0.016. In the Control and Hard GK test conditions, post-task ratings of knowledge and memory did not differ from the mid-point (all *t*s ≤ 2.09, *p*s ≥ 0.041). In sum, participants consistently rated their intelligence above average (assuming mid-point was perceived as average), whereas ratings of knowledge and memory were more variable.

**Table 2:** Mean (standard errors) ratings for memory, GK, and intelligence as a function of condition and time of assessment in Experiments 1 and 2.

		Easy GK test			Hard GK test			Control		
		Knowledge rating	Intelligence rating	Memory rating	Knowledge rating	Intelligence rating	Memory rating	Knowledge rating	Intelligence rating	Memory rating
Study 1	Pre-test	4.68 (0.12)	4.81 (0.13)	4.21 (0.15)	4.74 (0.13)	4.91 (0.13)	4.30 (0.19)	4.36 (0.14)	4.71 (0.13)	4.36 (0.17)
	Post-test	4.97 (0.01)	4.88 (0.15)	4.41 (0.17)	3.96 (0.17)	4.55 (0.17)	4.04 (0.20)	4.30 (0.15)	4.68 (0.14)	3.60 (0.19)
		*	n.s.	n.s.	*	*	*	n.s.	n.s.	*
Study 2	Pre-test	4.48 (0.10)	4.84 (0.12)	4.20 (0.15)	4.68 (0.13)	4.95 (0.14)	4.56 (0.17)	4.66 (0.13)	4.87 (0.13)	4.40 (0.17)
	Post-test	4.63 (0.13)	4.79 (0.15)	4.37 (0.16)	4.01 (0.16)	4.66 (0.17)	4.07 (0.19)	4.62 (0.13)	4.85 (0.14)	4.27 (0.18)
		n.s.	n.s.	n.s.	*	*	*	n.s.	n.s.	n.s.

Mean (standard errors) scores for self-rated knowledge, self-rated intelligence, and self-rated memory ability as a function of condition and time of assessment.

##### Knowledge ratings

Self-rated knowledge declined from pre- (*M* = 4.60, *SEM* = 0.08) to post-task (*M* = 4.41, *SEM* = 0.09), *F*(1, 194) = 8.10, *p* = 0.005, η_p_^2^ = 0.04, and ratings differed based on condition, *F*(2,194) = 4.50, *p* = 0.012, η_p_^2^ = 0.04. Participants in the Easy GK test condition gave higher ratings (*M* = 4.83, *SEM* = 0.13) than participants in the Hard GK test condition (*M* = 4.35, *SEM* = 0.13, *p* = 0.034) and those in the Control condition (*M* = 4.33, *SEM* = 0.14, *p* = 0.028). The latter two did not differ from one another, *p* > 0.99. The main effects were qualified by a significant interaction, *F*(2, 194) = 23.90, *p* < 0.001, η_p_^2^ = 0.20. Participants in the Control condition did not change their self-rated knowledge, *F* < 1.0, *p* = 0.599, whereas those in the Easy GK test condition increased it, *F*(1, 194) = 6.54, *p* = 0.011, η_p_^2^ = 0.03, and those in the Hard GK test condition decreased it, *F*(1, 194) = 49.66, *p* < 0.001, η_p_^2^ = 0.20. Thus, the difficulty manipulation was successful.

##### Intelligence ratings

Neither main effect was significant, both *F*s ≤ 3.73, *p*s ≥ 0.055; however, the interaction was, *F*(2, 194) = 5.84, *p* = 0.003, η_p_^2^ = 0.06. Participants in the Easy GK test and in the Control conditions did not change their assessments (both *F*s < 1.0, *p*s > 0.435), whereas participants in the Hard GK test condition gave lower assessments after answering the questions than before, *F*(1, 194) = 14.93, *p* < 0.001, η_p_^2^ = 0.07. This suggests that participants had an implicit idea that intelligence and knowledge are related, because poor performance on a GK test led them to revise their estimates of their own intelligence downwards whereas high performance on the easy GK test likely corroborated their expectations and did not impact their self-ratings of intelligence.

##### Memory ratings

Ratings of memory ability declined from pre- (*M* = 4.29, *SEM* = 0.10) to post-task (*M* = 4.02, *SEM* = 0.11), *F*(1, 194) = 16.55, *p* < 0.001, η_p_^2^ = 0.08. There was no main effect of task type, *F* = 1.0, *p* = 0.369. However, the interaction was reliable, *F*(2, 194) = 17. 41, *p* < 0.001, η_p_^2^ = 0.15. In the Easy GK test condition, ratings did not change from pre- to post-task, *F*(1, 194) = 3.00, *p* = 0.085, and ratings decreased in the Control and Hard GK test conditions, *F*(1, 194) = 4.91, *p* = 0.030, η_p_^2^ = 0.02, and *F*(1, 194) = 43.91, *p* < 0.001, η_p_^2^ = 0.18, respectively. The decrease in the Hard condition might reflect difficulty in recognizing the correct answer, which could serve as a proxy for memory ability. Specifically, if participants thought they knew the answers to the difficult questions but could not recognize them, they might attribute that difficulty to having a poor memory rather than a lack of knowledge. It is unclear why ratings in the Control condition changed, although the effect was small and might simply reflect measurement error.

#### Ratings of relationships between intelligence, knowledge, and memory

**Figure 2:**
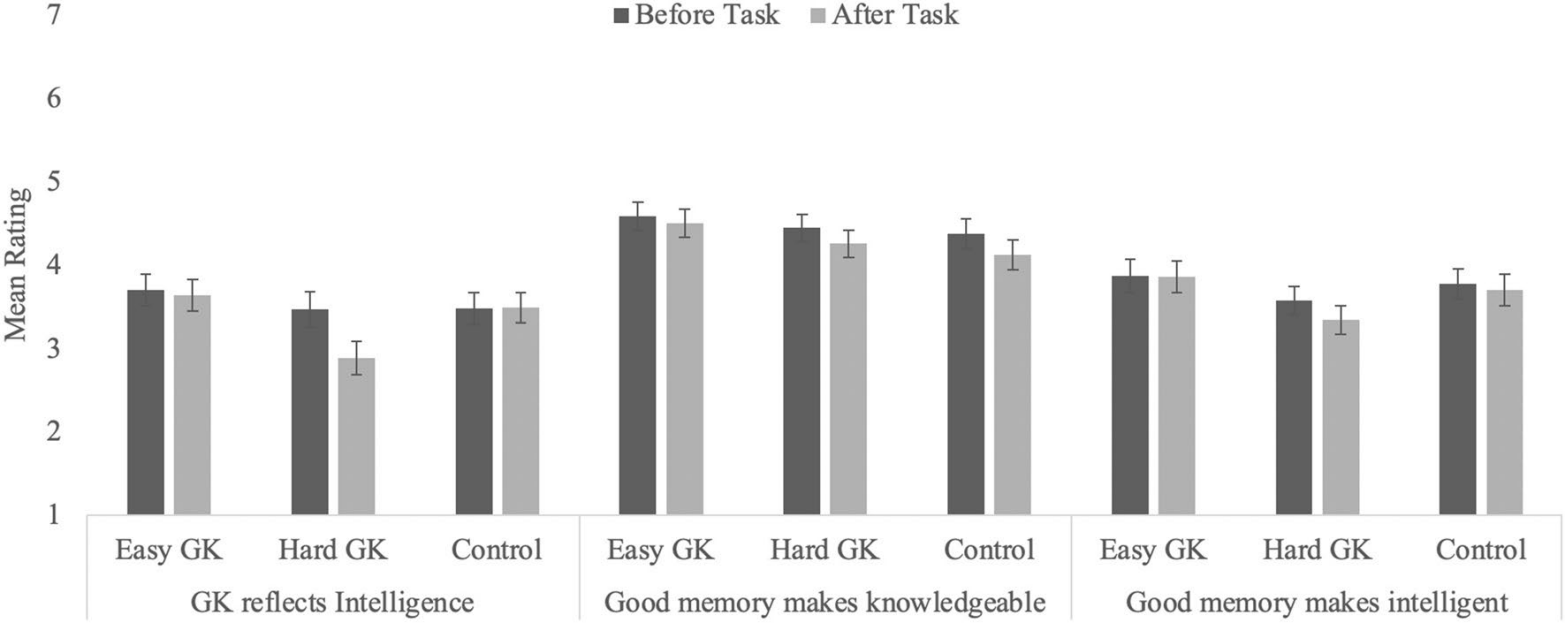
Average ratings for the perceived relationship between intelligence, knowledgeability, and memory in Experiment 1 as a function of condition and timing (error bars represent standard error of the mean).

Participants rated, before and after the interpolated task, the extent to which they thought being knowledgeable, being intelligent, and having a good memory depended on one another. We report these analyses next, using the same 2 × 3 mixed ANOVA (see Table 3 and Fig. 2). Because the Hard GK test affected participants’ ratings of all three constructs, these participants might also change their ratings of the relationship between constructs. Specifically, if poor performance on a GK test is perceived as a threat to one’s perceived intelligence, participants might reduce their estimates of the strength of the relationship between GK and intelligence.

**Table 3:** Mean (standard errors) ratings for the relationships between memory, GK, and intelligence as a function of condition and time of assessment.

		Easy GK test			Hard GK test			Control		
		GK and intelligence	GK and memory	Memory and intelligence	GK and intelligence	GK and memory	Memory and intelligence	GK and intelligence	GK and memory	Memory and intelligence
Study 1	Pre-test	3.70 (0.19)	4.59 (0.17)	3.87 (0.20)	3.47 (0.21)	4.45 (0.16)	3.58 (0.17)	3.48 (0.19)	4.38 (0.18)	3.78 (0.18)
	Post-test	3.64 (0.19)	4.51 (0.17)	3.86 (0.19)	2.89 (0.20)	4.26 (16)	3.35 (0.17)	3.49 (0.18)	4.13 (0.18)	3.70 (0.19)
		n.s.	–	–	*	–	–	n.s.	–	–
Study 2	Pre-test	3.50 (0.18)	4.43 (0.19)	4.06 (0.18)	3.52 (0.19)	4.71 (0.18)	4.09 (0.20)	3.66 (0.18)	4.52 (0.18)	3.96 (0.18)
	Post-test	3.57 (0.19)	4.41 (0.19)	3.96 (0.19)	3.31 (0.19)	4.40 (0.16)	3.94 (0.18)	3.75 (0.17)	4.40 (0.18)	3.84 (0.16)
		–	–	–	–	–	–	–	–	–

Because the interactions were not significant, no follow-up tests were conducted as a function of condition other than to examine the relationship between intelligence and GK.

##### Relationship between intelligence and knowledge

The effects of timing and the interaction were significant, *F*(1, 194) = 8.23, *p* = 0.005, η_p_^2^ = 0.04, and *F*(2, 194) = 6.47, *p* = 0.002, η_p_^2^ = 0.06, respectively. Overall, ratings decreased from pre- (*M* = 4.55, *SEM* = 0.11) to post-task (*M* = 3.34, *SEM* = 0.11). However, whereas participants in the Easy GK test and Control conditions did not change their responses (both *F*s < 1.0, *p*s ≥ 0.641), participants In the Hard GK test condition gave lower ratings on the post-task assessment than on the pre-task assessment, *F*(1, 194) = 21.54, *p* < 0.001, η_p_^2^ = 0.10. The effect of task type was not significant, *F*(2, 194) = 1.87, *p* = 0.157. Thus, when participants struggled to respond to difficult GK questions, they changed their ratings to serve a protective function: Intelligence and knowledge were considered less strongly related.

##### Relationship between memory and knowledge

Only the effect of timing was significant: Participants across all three conditions gave lower ratings post-task (*M* = 4.30, *SEM* = 0.10) than pre-task (*M* = 4.48, *SEM* = 0.10), *F*(1, 194) = 5.67, *p* = 0.018, η_p_^2^ = 0.03. The other effects were not significant, *F*s < 1.0, *p*s ≥ 0.41.

##### Relationship between memory and intelligence

Finally, the perceived relationship between intelligence and memory was unaffected by task type or timing, all *F*s ≤ 3.32, *p*s ≥ 0.070. This is not surprising, considering neither the control task nor the GK task were traditional memory or intelligence tasks and thus were not expected to influence one another strongly.

#### GK test performance and self-ratings

To examine whether objective performance on the GK test influenced self-assessments of knowledge and intelligence, we performed a median split of GK test performance to identify high- and low-scoring individuals within each condition. We then ran an ANOVA with timing, task type, and performance group as factors. According to the Dunning-Kruger effect^37^, the worst performers should be the most likely to overestimate their abilities, especially prior to performing the task.

For the self-assessments of knowledge, objective performance interacted with timing, *F*(1, 131) = 5.31, *p* = 0.001, η_p_^2^ = 0.07: Regardless of GK condition, participants who performed poorly assessed their knowledge lower after the GK test than before the test; those who performed well did not change their assessments. Thus, objectively less knowledgeable participants were able to re-evaluate their knowledge given the feedback and relative comparison to the normative group. Regarding the self-assessments of intelligence the three-way interaction was significant, *F*(1, 131) = 5.63, *p* = 0.019, η_p_^2^ = 0.04. Participants in the Easy GK condition did not alter their assessments of intelligence; in contrast, lower performing participants in the Hard GK condition rated their intelligence lower after the test, but higher performing participants did not, *F*(1, 66) = 8.87, *p* = 0.004, η_p_^2^ = 0.12.

### Discussion

Participants decreased their self-rated intelligence and memory after the hard GK test; however, scores following the easy GK test remained the same. Participants decreased their self-rated knowledge following a hard test and increased it following an easy test, indicating they perceived their performance on the test as an accurate reflection of how knowledgeable they were. The latter served, in part, as a manipulation check to show that laypeople believe that GK does measure the concept of knowledge/knowledgeability. Furthermore, participants adjusted their estimates in a way that accounted for high or low- performance levels, reflecting relatively accurate metacognitions.

In terms of construct relatedness, after a hard GK test, participants were less inclined to believe that GK is a good indicator of intelligence. Conversely, an easy GK test did not affect the relationship. Finally, participants did not change their view on the relationship between memory and knowledge or memory and intelligence after either GK test.

The finding that participants decreased their ratings of the relatedness between knowledge and intelligence after the hard GK test suggests that people tend to believe that tasks they excel at are accurate indicators of their abilities^19^, and that they strive for a positive self-concept^5,6^. To maintain the belief that one is intelligent despite poor performance on a cognitive task—in this case, a GK test—they must conceptualize intelligence in a way that de-emphasizes GK’s role.

Interestingly, participants also decreased their self-ratings of intelligence. This suggests that the task diminished their beliefs about their own intelligence to an extent. This finding somewhat contradicts our conclusion regarding participants’ ability to preserve their beliefs about their intelligence. In other words, if participants do not believe GK is a good measure of intelligence, they should not decrease their self-ratings of intelligence after performing poorly on a difficult GK test. Thus, how one conceptualizes the relationship between these constructs and their performance is complex, and participants might not have clear insights into their own processes. We return to this point in the General Discussion.

Individuals approach cognitive tasks differently depending on their implicit theories of intelligence^17,28,38–42^. In educational settings, those who endorse an entity theory of intelligence emphasize the performance of intelligence^43^. These students believe they must prove that they are intelligent by achieving high grades and bolstering their academic standings relative to others. The instructions for the GK test may have primed participants to approach the task with this mindset. We told participants that 50% of people could answer the questions correctly, and that we were interested in what the average person knows. If participants approached the task with a desire to prove their intelligence through the GK test, the decreased self-ratings of intelligence after poor test performance might indicate they assumed their poor performance was diagnostic of their abilities^44^.

One question is to what extent participants’ responses were affected by the initial manipulation. By providing the normative (mis)information, we created a situation in which participants were given an objective (albeit incorrect) anchor against which to compare themselves. Thus, the changes in ratings could be due to the fact that they received feedback that they were, in fact, more or less intelligent than the average person, or that they were relying on an internal signal or metacognitive response caused by the effort or lack thereof in retrieving the answer.

## Experiment 2

The simple act of attempting to answer obscure GK questions may lead participants to alter their assessments, even in the absence of any information about normative performance. To examine this, in Experiment 2, we tested a new sample of participants and removed the deception: participants completed the same tasks, but the GK test groups were not provided any (mis)information about how the average person performed. If effort or ease in recognizing the correct answer is used as an indicator of one’s knowledge, memory, or intelligence, we would expect to observe similar results. Conversely, if the assessment process is only affected by a comparative judgment in which one evaluates one’s own performance relative to an “average” person, the absence of any such information should lead participants to not change their responses.

### Method

#### Participants

Two hundred twelve participants from the same source as Experiment 1 were recruited. Ten revoked consent and two timed out, leaving 200 valid data sets (117 women, our provided no information). The average age was 23.09 (*SD* = 3.37, range 18–31) and the average completion time was 7.41 min (*SD* = 5.09, range = 1.44–40.90). The average compensation was $12.77/h.

#### Materials and procedure

The experiment was identical to Experiment 1, except that the participants in the GK test conditions were not provided any information about the population-level performance. Accuracy feedback was still provided, so participants would know about their general performance level.

### Results

#### Analysis overview

**Figure 3:**
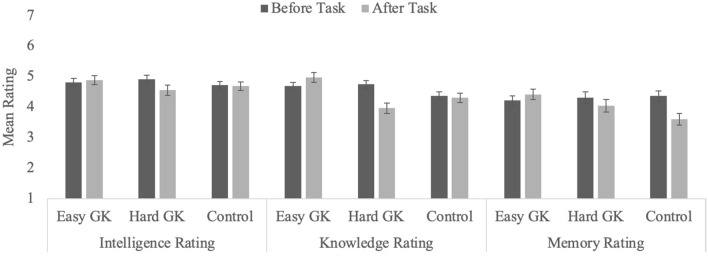
Average ratings for self-assessment of intelligence, knowledgeability, and memory in Experiment 2 as a function of condition and timing (error bars represent standard error of the mean).

We conducted the same analyses as in Experiment 1 (see Table 1 for descriptive statistics). Data from four participants (three in the Easy GK condition and one in the Hard GK condition) were excluded because they responded “yes” to the question about searching for answers online. Thus, analyses included 65 participants in the Easy GK condition, 64 in the Hard GK condition, and 67 in the Control condition (see Table 2 and Figure 3).

Again, participants correctly answered more questions in the Easy GK condition (*M* = 0.95, *SEM* = 0.008) than in the Hard GK condition (*M* = 0.25, *SEM* = 0.02), *t*(90.09) = 34.60, *d* = 6.00.

#### Self-ratings of intelligence, knowledge, and memory

##### Overall ratings

Average ratings are in Table 1 and Fig. 2. At the pre-test, participants in all three conditions rated their knowledge and intelligence above the mid-point, all *t*s ≥ 4.59, *p*s ≤ 0.001. Self-rated memory did not differ from the mid-point in the Easy GK and Control conditions (*t*s ≤ 2.37, *p*s ≥ 0.021). In the Hard GK condition, participants did rate their memory above the mid-point, *t*(66) = 3.28, *p* = 0.002. There were no differences across conditions in ratings (all *p*s ≥ 0.304). Post-test, self-rated intelligence was still above the mid-point in all three conditions (all *t*s ≥ 3.75, *p*s ≤ 0.001), and knowledge ratings were above four in both the Easy condition and the Control condition, *t*s ≥ 4.64, *p*s ≤ 0.001, but not in the Hard condition (*t* < 1.0, *p* = 0.957). Post-test ratings of memory did not differ from the mid-point (all *t*s ≤ 2.25, *p*s ≥ 0.028). The results are largely consistent with those of Experiment 1: participants consistently rated their intelligence above average^45^. Knowledge and memory ratings tended to vary more, and, in some cases, not differ from the mid-point.

##### Knowledge ratings

Participants’ ratings of their knowledge decreased from pre- (*M* = 4.61, *SEM* = 0.07) to post-task (*M* = 4.42, *SEM* = 0.08), *F*(1, 193) = 8.91, *p* < 0.001, η_p_^2^ = 0.04. The effect of task type was not significant, *F*(2, 193) = 1.56, *p* = 0.21, but the interaction was, *F*(2, 193) = 15.84, *p* < 0.001, η_p_^2^ = 0.14. Whereas participants in the Easy GK and the Control conditions did not change their ratings (both *F*s ≤ 1.83, *p*s ≥ 0.178), participants in the Hard GK condition reduced their ratings from pre- to post-task, *F*(1, 193) = 39.21, *p* < 0.001, η_p_^2^ = 0.17. Thus, mere exposure to difficult and unfamiliar GK questions led participants to adjust their assessment of their own knowledge. However, in the absence of the misleading information about others’ performance, participants in the Easy GK condition did not increase ratings of their own knowledge.

##### Intelligence ratings

The effect of timing was significant, *F*(1, 193) = 5.80, *p* = 0.017, η_p_^2^ = 0.03, such that participants lowered their ratings from 4.89 (*SEM* = 0.08) to 4.76 (*SEM* = 0.09), as was the interaction, *F*(2, 193) = 3.07, *p* = 0.049, η_p_^2^ = 0.03. The effect of task type was not reliable, *F* < 1.0, *p* = 0.958. Follow-up tests revealed the same pattern as in Experiment 1: No change for participants in the Easy GK and Control conditions (both *F*s < 1.0, *p*s ≥ 0.601), and a significant decline in ratings for those in the Hard GK condition, *F*(1, 193) = 11.93, *p* = 0.001, η_p_^2^ = 0.06. This suggests that difficulty answering GK questions does affect one’s perceived intelligence, even if participants are not provided (mis)information about normative performance.

##### Memory ratings

The ANOVA revealed a significant effect of timing, *F*(1, 193) = 7.40, *p* = 0.007, η_p_^2^ = 0.04, no effect of task type, *F* < 1.0, *p* = 0.976, and a significant interaction, *F*(2, 193) = 11.37, *p* < 0.001, η_p_^2^ = 0.10. There were no changes in assessments for participants in the Easy GK and Control conditions (both *F*s ≤ 2.79, *p*s ≥ 0.097), but a significant drop in ratings for participants in the Hard GK condition, *F*(1, 193) = 25.90, *p* < 0.001, η_p_^2^ = 0.12. The stability observed in the Control condition suggests that the effect observed in Experiment 1 was a random error.

#### Ratings of relationships between intelligence, knowledge, and memory

##### Relationship between intelligence and knowledge

**Figure 4:**
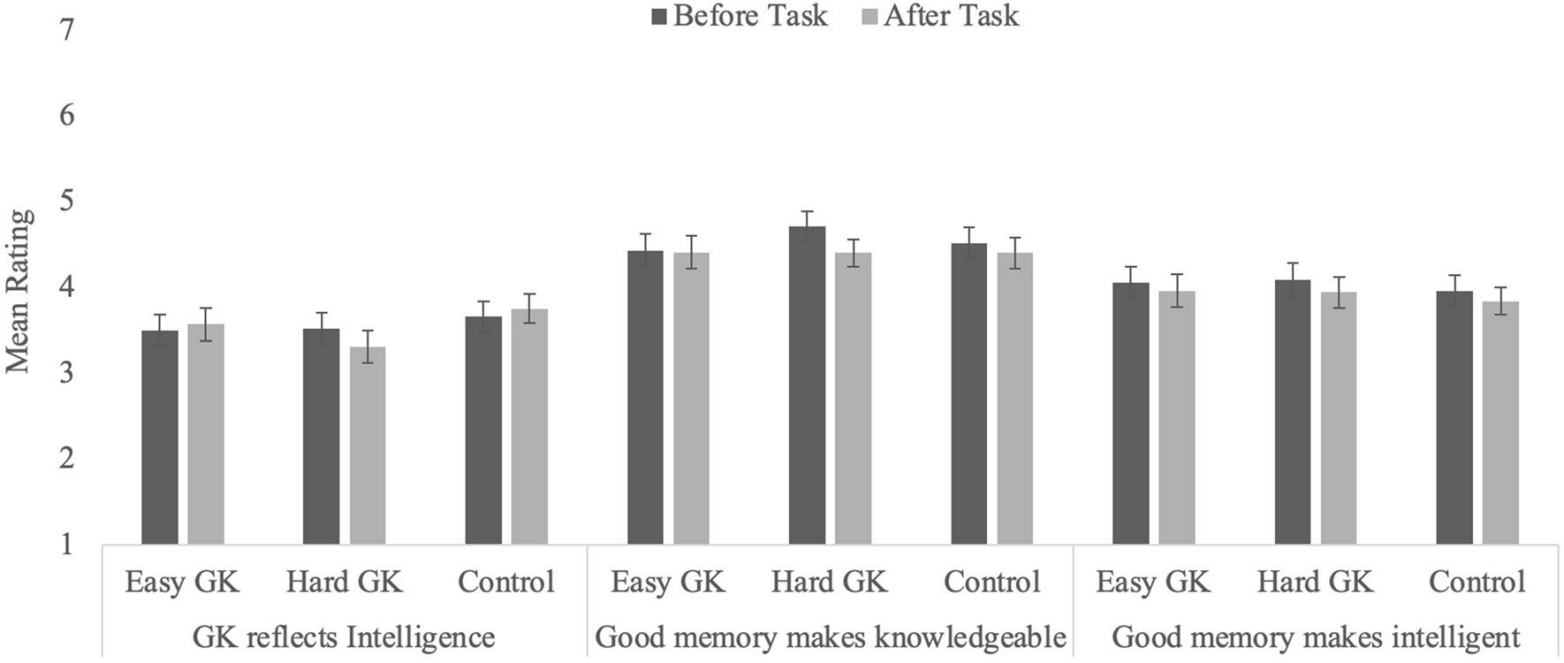
Average ratings for the perceived relationship between intelligence, knowledgeability, and memory in Experiment 2 as a function of condition and timing (error bars represent standard error of the mean).

None of the effects were significant, all *F*s ≤ 1.78, *p*s ≥ 0.17 (Fig. 4). Thus, the change in the association between knowledge and self-rated intelligence appears to be due to the manipulation in Experiment 1: When participants had no expectation of their performance relative to the general population, they did not need to engage in any self-preservation.

##### Relationship between memory and knowledge

As in Experiment 1, ratings of the strength of the relationship between knowledge and memory declined from pre- (*M* = 4.55, *SEM* = 0.10) to post-task (*M* = 4.40, *SEM* = 0.10), *F*(1, 193) = 5.76, *p* = 0.017, η_p_^2^ = 0.03. Neither the interaction nor the main effect of condition was significant, both *F*s ≤ 2.14, *p*s ≥ 0.12.

##### Relationship between intelligence and memory

Finally, and consistent with Experiment 1, none of the effects were significant in the analysis examining the relationship between intelligence and memory (*F*s ≤ 3.23, *p*s ≤ 0.074).

#### GK test performance and self-ratings

As in Experiment 1, we conducted median split analyses based on GK Test performance. The differential re-assessment of abilities observed in Experiment 1 did not occur in Experiment 2 (all effects and interactions involving the performance variable *p*s ≥ 0.179).

### Discussion

The results of Experiment 2 partially replicate those of Experiment 1: after attempting to answer hard GK questions, participants lowered their estimates of their own abilities in all three constructs. Without misleading information, participants likely relied on their experience of difficulty and the feedback to determine their ability. More interestingly, without (mis)information, there was no change in how participants rated the relationship between intelligence and knowledge. Thus, normative (mis)information appears to exert distinct effects on the extent to which one might need to engage in a self-preservation process. The analyses based on objective performance in the GK test, indicated that participants relied on what was perhaps the most salient bottom-up cue: recognition difficulty. Interestingly, this did not affect participants’ overall rating of their own memory but just the extent to which memory was related to knowledge. It is possible that the other two constructs—knowledge and intelligence—are considered more important or valued more centrally and thus require more compensatory behaviors.

## General discussion

### Summary of main findings

Across both experiments, taking a hard GK test led participants to reduce the estimates of their own knowledge, intelligence, and memory. This occurred regardless of whether participants were misled to believe that the answers were known by about 50% of the general population, suggesting that recognition difficulty and correct answer feedback were driving the effect. Participants only altered their ratings about how strongly knowledge and intelligence are related when provided with the normative (mis)information. Specifically, participants believed the link between intelligence and knowledge was weaker after inferring that their knowledge was below average. Because the GK test is a measure of crystallized intelligence, it confirms that participants’ understanding of intelligence does incorporate knowledge as a core component^26^ and that threats to one’s standing relative to an imaginary normative group trigger a protective response. Critically, a basic finding is that perceptions of their own intelligence, memory, and knowledge are malleable following a brief GK task.

### Effects on perceptions of intelligence

Cognitive dissonance theory may explain why participants decreased their self-ratings of intelligence while also decreasing the extent to which they believe that GK reflects intelligence in Experiment 1^46^. People tend to seek consistency and reduce the dissonance between contradictory cognitions. By approaching the GK test believing that their performance will demonstrate their above-average intelligence and then performing poorly on said test, one is forced to recognize a contradiction. On the one hand, they believe they possess above-average intelligence; on the other hand, their performance on the GK test reflects lower intelligence. One way to reduce dissonance is to rationalize the event by adding a third notion, making the original two differing cognitions appear more consistent^47^. Here, reducing the extent to which one believes that GK is a good indicator of intelligence enables one to feel that they are indeed intelligent alongside their admission that they are not as intelligent as they initially thought. This only occurred when participants were given false normative information, which might have activated prior beliefs about relative abilities and one’s ranking among comparison groups. As noted previously, whereas lowering the ratings of the perceived relationship between intelligence and GK could serve a protective function, lower ratings of one’s own intelligence would create a threat to the self-concept. A potential explanation is provided by the finding that lay participants’ definitions of intelligence tend to include knowledge as a core dimension^26^. Although speculative, it is possible that participants reduction of their own intelligence was intended to reflect selective reductions in crystallized intelligence, leaving fluid intelligence intact. Future work might examine more nuanced measures of intelligence, for example, clearly distinguishing fluid and crystallized.

### Effects on perceptions of knowledge and memory

Participants in the Hard GK condition decreased their self-ratings of knowledge after receiving confirmatory evidence that they were not as knowledgeable as they initially thought. In both experiments, participants also decreased their self-rated memory after taking the difficult GK test, suggesting they might have attributed their poor performance to recognition failures rather than a total lack of knowledge. In Experiment 1, the (mis)information manipulation implied that a majority of people know (and could retrieve) the information necessary for accurately answering the questions on the GK test. Because participants generally rated their knowledge as above average before the test, they might believe that they should know more than the average person and perform well on the test. This prediction assumes that one has been exposed to the same or more information than others; thus, one might attribute their poor performance on the difficult GK test to an inability to retrieve the necessary information, not to a lack of knowledge.

However, similar results occurred in Experiment 2. A more critical factor might, then, be the experience of recognition difficulty during the GK test. Participants likely struggled to search for the information or experienced uncertainty. The phenomenological state of *not remembering* is characterized by the feeling that one was exposed to the information at some point but cannot retrieve it on command. Conversely, the state of *not knowing* is defined by a sense that one was never exposed to the information in question^36^. If the question felt familiar or participants felt they “should have known it,” this might have led them to experience a state of *not remembering*, which, in turn, could be attributed to poor memory rather than a lack of knowledge. Familiarity with a question or retrieval of related information can increase the feeling of knowing^48^, and a similar process likely occurs when participants are attempting to retrieve information and can access related or incomplete knowledge^49^. Although the questions were challenging, the multiple-choice format likely increased the perceived familiarity by providing additional contextual information. Indeed, our results show that participants’ beliefs about the relatedness between memory and knowledge remained stable from before to after taking the difficult GK test. However, we note that although the overall relationship between memory and the other two constructs showed stability in Experiment 2, it did appear that those participants who performed the worst on the GK test did lower their estimates of the relationship between constructs. Perhaps, the experience of difficulty in recognizing the correct answer with no additional information about normative performance directed participants’ attention to the role of retrieval processes. Together, these findings suggest that participants believe the extent to which one is knowledgeable depends at least in part on their ability to access information.

### Feedback and comparative judgments

Self-ratings of one's mental abilities and conceptualizations of intelligence are easily influenced, at least in the short term, by feedback about one's performance on a cognitive task^10,12,16,20^. Participants in both experiments were given accuracy feedback, thus allowing a direct and immediate assessment of performance, and participants in Experiment 1 were given (mis)information concerning their performance relative to the general population. Relative to others or not, participants rated themselves lower on the constructs assessed here when given feedback that indicated poor performance. Here, participants used the feedback to modify their self-assessments in a way that is adaptive: poor performance on a GK task should elicit adjustments to one’s perceived level of knowledge.

Providing participants with (mis)information that conveyed their success on the easy GK test relative to others increased assessments of their knowledge. In the absence of normative (mis)information, however, the easy GK test did not affect knowledge ratings. This underscores how sensitive participants seem to be to relative comparisons. This type of information may have negative consequences for learning. Dweck^50^ theorized that praise for success causes individuals to seek out other tasks where they believe success is guaranteed, while avoiding opportunities to learn and grow through failure^28^, contrary to the assumption that praise for success would cause one to seek out challenging learning tasks in the future. Our results suggest that the ease of the task may superficially inflate participants' gains in confidence in their mental abilities and be conducive to motivational goals of seeking out success relative to others while avoiding learning opportunities that entail a risk of failure.

More generally, these results highlight the interactive roles of top-down beliefs about one’s abilities and self-concept and the bottom-up information conveyed by effort, familiarity, and feedback^18^. Top-down processes, here, refer to individual characteristics of the learner, their self-evaluations and assessments, and include beliefs about one’s abilities, personal metacognitive knowledge, self-efficacy, and so on. These beliefs can be conscious or implicit and are initially relatively stable. Bottom-up influences are task-derived and vary across situations or scenarios as one assesses their performance. Feelings of effort, difficulty, or fluency provide feedback and affect performance evaluation^47,51^. Furthermore, individuals can update their self-concept in the relevant domain^52^ and eventually their self-assessments. Jointly, top-down and bottom-up processes impact performance and metacognition^29^.

Participants clearly hold beliefs and expectations about their skills in a given task or domain, which guide behavior and performance evaluations. An adaptive system needs to incorporate new information (e.g., feedback) and phenomenological cues (e.g., familiarity, fluency of processing) to accurately guide current and future behavior^51,53^.

### Addressing metacognitive errors

Although not a primary goal, our results have implications for some common metacognitive errors. The better-than-average effect^54^ refers to the fact that across a variety of abilities, people tend to claim to be “better than the average person” at a task. This effect occurs across ages and domain abilities, even for implausible and highly skilled tasks, such as landing an airplane [^55^]. The need for self-enhancement (i.e., the claim to be above average) is more likely to emerge for domains and abilities that individuals might consider more central, important, or easy^34^. Ratings above the midpoint were most consistent in the domain of intelligence. Relative to memory or knowledge, intelligence might be perceived as a more important domain upon which individuals develop their self-worth.

In Experiment 1, participants who were objectively less knowledgeable in the Hard GK condition (i.e., participants who scored in the bottom half in terms of accuracy) lowered their self-ratings of knowledge. Upon receiving feedback in terms of performance relative to a normative group, it would be beneficial for assessments of one’s knowledge and intelligence to be re-adjusted, especially if one performed particularly poorly. Thus, this adjustment is potentially adaptive. In the Easy GK condition, re-evaluations of knowledge also occurred, with higher performers increasing their estimates. Such results are suggestive of reasonably accurate metacognitive awareness, in contrast to the typical Dunning-Kruger pattern of results^37^. Indeed, this is consistent with prior work showing that participants can be sensitive to material’s difficulty^56^ as well as other factors like time pressure in terms of their study time allocation^57,58^. In the Hard GK condition, a similar finding occurred for intelligence ratings as for knowledge ratings: Individuals who performed more poorly lowered their ratings. However, these patterns did not occur in the absence of the normative (mis)information, underscoring the importance of the normative comparison in how participants re-evaluate their abilities.

Therefore, under some conditions, low performing individuals can and do re-evaluate their assessments of their own abilities, and they do so in a way that reflects actual performance. Thus, low performance on a task does not necessitate poor metacognition—here, they are decoupled. However, these reassessments seem to depend on the presence of some sort of comparison group or information about how others typically perform.

### Implications

These findings have implications for educators seeking to promote learning and adaptive motivational goals for all students. Educators may want to avoid the common practice of reporting class averages for tests because the awareness that one performed poorly relative to others may cause students to lower their perceptions of their own abilities. This might be especially important for students from marginalized groups or who perceive educational settings as threatening^59,60^.

A reduction in how one perceives one’s abilities may also lead to adopting motivational goals directed toward avoiding failure and seeking out easy success. Individuals who believe in their academic abilities and are more motivated to work hard tend to have higher grade-point averages (GPAs^61^). Thus, educators may wish to avoid enforcing educational practices that promote social comparison in order to foster students' reliance on their own abilities and encourage hard work. People seem to recalibrate quite readily based on contextual information, such as the normative (mis)information and feedback provided in Experiment 1. However, at least at the short term, these practices may harm self-perceptions of ability. Interventions might target this malleability and promote more adaptive and accurate metacognition. In other words, adjusting one’s beliefs and perceptions in response to evidence about how one performed could be adaptive if it leads to behavioral changes (e.g., poor performance on an assessment could lead to more effective studying).

### Limitations and future directions

The present work has limitations that lead to future avenues of research. First, our samples likely represent a large but unmeasured range of participant perspectives on the importance and value of their intelligence, knowledge, and memory to their self-worth. Future research may do well to examine shifts, or lack thereof, in these self-perceptions in those for whom these concepts are more or less critical to their sense of self-worth. Second, though our findings provide evidence that self-perceptions of intelligence, knowledge, and memory are malleable, the observed change in ratings was essentially immediately after the general knowledge test. Understanding the longevity of such effects using a longer delay before re-assessment is important for boosting positive outcomes and mitigating negative consequences. Third, the main data are self-ratings of these concepts and their relationships. Participants’ understandings and lay theories of these concepts (and the degree to which they map onto the scientific perspective) may be needed to illuminate some patterns observed. Furthermore, no downstream consequential measures were included. Will these changes in self-ratings translate to changes in behavior? Future studies could examine the extent to which task or test difficulty results in behavioral changes, such as selecting easier or more challenging activities. Finally, we acknowledge that our perspective, and that of our participants, is grounded in Western and white-dominant cultural frameworks. Thus, whether our findings would extend to more diverse samples and to groups with different cultural backgrounds remains an open and critical question.

## Conclusions

To conclude, performance on a GK test affects people's understanding of the relatedness between memory, knowledge, and intelligence, as well as their self-ratings of their abilities and these perceptions are easily subject to change. Moreover, ratings of one’s knowledge and intelligence are also subject to change, underscoring the complexity of these constructs. Although the manipulation was simple and the delay between measures was short, repeated cues that one is not as knowledgeable as others could have a cumulative effect on how one perceives one’s intelligence. Small yet frequent reminders of one’s lack of knowledge could give rise to feelings such as imposter syndrome rather than providing useful metacognitive information meant to adjust efforts, etc. In other words, the malleability of these constructs can be beneficial if it promotes adjustment and changes, such as improved study habits. However, there is also a potential for harm if individuals internalize implied messages about their inadequacy and are unable to counteract them. Future research needs to further explore such potential mechanisms that could lead to attrition or drop-out rates among students.

## Data Availability

Data are available on the first author’s website: https://web.colby.edu/memoryandlanguagelab/publications/stimuli-and-data-sets/.
